# New chamber stapes prosthesis – A preliminary assessment of the functioning of the prototype

**DOI:** 10.1371/journal.pone.0178133

**Published:** 2017-05-23

**Authors:** Monika Kwacz, Magdalena Sołyga, Maciej Mrówka, Konrad Kamieniecki

**Affiliations:** 1 Warsaw University of Technology, Institute of Micromechanics and Photonics, Warsaw, Poland; 2 Warsaw University of Technology, Institute of Radioelectronics, Warsaw, Poland; 3 Institute of Physiology and Pathology of Hearing, Kajetany, Nadarzyn, Poland; Mersin Universitesi Tip Fakultesi, TURKEY

## Abstract

Piston-stapedotomy is the most common method for hearing restoration in patients with otosclerosis. In this study, we have experimentally examined a prototype of a new chamber stapes prosthesis. The prototype was implanted in a human cadaver temporal bone. The round window vibrations before and after implantation were measured for the acoustic signal (90 dB SPL, 0.8–8 kHz) in the external auditory canal. In comparison with a 0.4-mm piston prosthesis, the chamber prosthesis induced significantly higher vibration of the round window, especially for frequencies above 1.5 kHz. Based on the results, it can be surmised that stapedotomy with a chamber stapes prosthesis could provide better hearing results in comparison with the piston-stapedotomy.

## Introduction

Piston stapes prostheses are commonly implanted in patients suffering from conductive or mixed hearing loss caused by stapes otosclerosis. In otosclerosis, mobility of the stapes is significantly reduced by abnormal bone growth in the oval window (OW) niche, or by stiffening of the stapes annular ligament (AL). The prosthesis stimulates the inner ear and restores hearing, with varying degrees of success. Piston-stapedotomy using the small-fenestra technique is a standard procedure, as it is minimally invasive, relatively safe and efficient [1–6]. During stapedotomy, the suprastructure of the otosclerotic stapes is removed, a small hole is drilled in the immobile stapes footplate (SF), the piston is introduced into the hole and the prosthesis is attached to the long process of the incus. Typical stapes prosthesis consists of a cylindrical piston and an attachment, usually a wire or ribbon, designed to be crimped around the incus. Prostheses currently in use differ in the piston diameter, type of material, and method of attachment to the incus. The piston diameter is between 0.4 mm and 0.8 mm [7, 8].

Postoperative hearing results are commonly reported following the American Academy of Otolaryngology-Head & Neck Surgery (AAO-HNS) guidelines [9]. Auditory outcomes are generally perceived as good but only for low and medium frequencies (0.5–3kHz), and a large number of patients report a lack of satisfactory results for higher frequencies [2, 4–6]. Wysocki et al. [10] have experimentally shown that the piston prosthesis leads to ~14 dB lower perilymph vibration at the round window (RW) compared to the physiological situation, in particular for frequencies above 2 kHz. Sim et al. [11] reported similar experimental results, which were based on difference in the RW volume displacement between a normal and reconstructed ear. The results of vibration measurements in temporal bones correlate to air bone gaps (ABGs) in patients after stapes surgery.

The postoperative hearing results are closely related to the smaller piston area compared to the normal SF area. The reduced area leads to decreased fluid volume displacement (VD) at the OW. Predictions using a simple lumped element model showed that the smaller the piston diameter the greater the ABG [12]. Additionally, (1) clinical observations [13–15], (2) finite element modelling [16–19], and (3) experiments on cadaver temporal bones [10, 11, 20] showed that stapedotomy with a piston prosthesis significantly changes middle-ear biomechanics and affects the residual ABG.

Prosthesis malfunction (displacement from a small hole in the SF or incus, immobilization by adhesion or fibrous tissue, necrosis or erosion of the long process, bony regrowth in the OW) causes the reappearance of conductive hearing loss and requires revision stapes surgery. Lesinski [21, 22] reported that 81–87% cases of revision-stapedotomy demonstrate a displaced or malfunctioning prosthesis. It is generally recognized that revision surgery provides less satisfactory hearing results than the primary operation [23, 24]. Moreover, revision stapes surgery is technically more difficult and has a higher rate of complications [22, 25, 26]. Following revision stapedotomy, the incidence of vertigo and/or sensorineural hearing loss is the most probable complication. Despite the aforementioned disadvantages, particularly the limited hearing improvement and prosthesis malfunction, piston prostheses are the only ones available for clinical use.

The aim of this study is to answer the following questions: (1) Is it possible to build a new stapes prosthesis that mimics the natural ear according to the invention described in [27]? (2) Does this new prosthesis efficiently transmit vibration for frequencies from 0.4 to10 kHz? To this end, we have designed and manufactured a prototype of a new chamber stapes prosthesis (ChSP). This prototype was implanted in a human temporal bone and its functioning was assessed based on vibration measurements.

## Methods

### Design of the ChSP prototype

**Fig 1:**
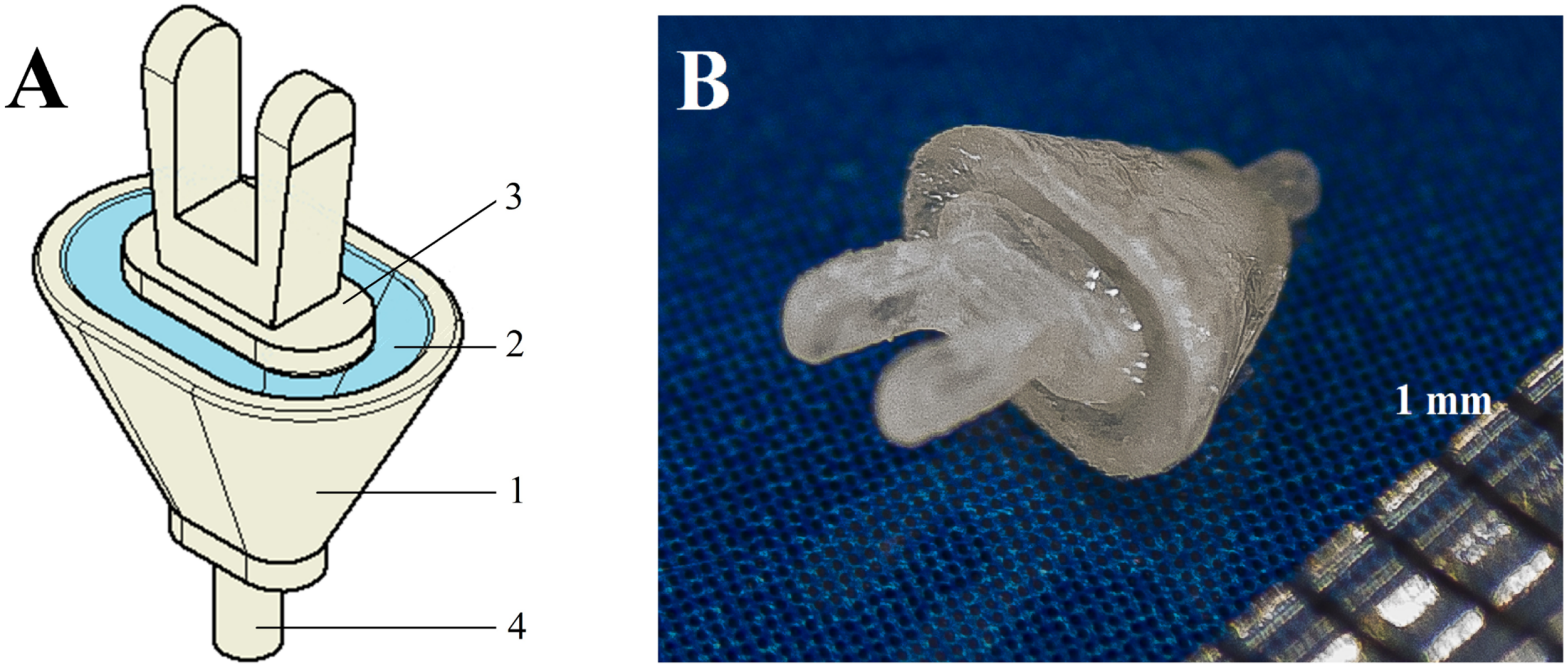
Chamber stapes prosthesis. (A) Design of the ChSP. 1 –conical chamber filled with fluid, 2 –flexible membrane (mimics the AL), 3 –rigid plate with an attachment (mimics the stapes), 4 –thin capillary tube (connects the chamber with the vestibule and allows fluid to flow). (B) Photo of the ChSP prototype filled with fluid.

Our prototype ChSP [see Fig 1(A)] consists of: (1) a conical chamber mimicking the vestibule, (2) a flexible membrane mimicking the annular ligament, and (3) a rigid plate mimicking the stapes. The conical chamber is filled with fluid (in this study we used deionized water) and covered with a flexible membrane. The chamber ends with a thin tube (4) designed to insert into a hole made in the SF. Through the open end of the tube, the fluid merges with perilymph, filling the natural vestibule. The rigid plate with an attachment is fixed to the membrane. For the attachment, we designed two parallel walls between which the incus long process is inserted and fixed using glass-ionomer cement. After attaching the plate to the incus, incus vibrations are transmitted to the fluid. Motion of deionized water inside the chamber is transferred to the cochlear vestibule through the open end of the thin tube. The ChSP functioning has been numerically simulated [19] and shows that BM responses in the cochlea with the new prosthesis are higher, compared to the healthy ear. In this study, a prototype ChSP was made and was experimentally implanted in a human cadaveric temporal bone specimen. To assess the ChSP functioning, vibration of the rigid plate and the RW membrane were measured.

Both the shape and dimensions of the ChSP prototype were established based on anthropometric data published in literature, and on our earlier micro-CT measurements [28, 29]. The area of the large chamber base and rigid plate is 6.7 mm^2^ and 2.8 mm^2^, respectively. Part (4) of the chamber is formed as a thin capillary tube (OD = 0.5mm, ID = 0.3mm). The chamber and rigid plate were made using rapid prototyping technique (ProJet 3D Printer, Potomac Photonic Inc, Halethorpe, MD 21227, USA) based on the 3D CAD models (Autodesk Inventor^®^ 3D). Both parts were made from ABS (Acrylonitrile-Butadiene-Styrene). The 0.3-mm ID and high value ABS surface tension (~38.5 mJ/m^2^) ensure that the fluid does not flow out through the open end of the tube during surgical manipulation.

The flexible membrane was self-made according to technique described by Olson [30] for a diaphragm dedicated to an intracochlear pressure sensor. We have used a liquid UV-light curing adhesive NOA 68 (Norland Optics, New Brunswick, NJ, USA). A drop of NOA 68 adhesive was placed on the surface of deionized water. After spreading into a thin film, it was pre-cured with UV light (36 W) for 25s. The thickness of the pre-cured membrane was estimated at hundreds of nanometers, based on the interference-induced coloration of the film [30]. The chamber was then placed in the vessel, under the water surface. After the water filled the chamber, the floating pre-cured membrane was scooped up by the chamber held in tweezers. The rigid plate was placed on the pre-cured membrane, closing the chamber from the top. When all components had been assembled, the membrane was cured with UV light for an additional 10 minutes. After curing, the membrane appeared thicker, but it thickness is still only 20–30 micrometers.

**Fig 2:**
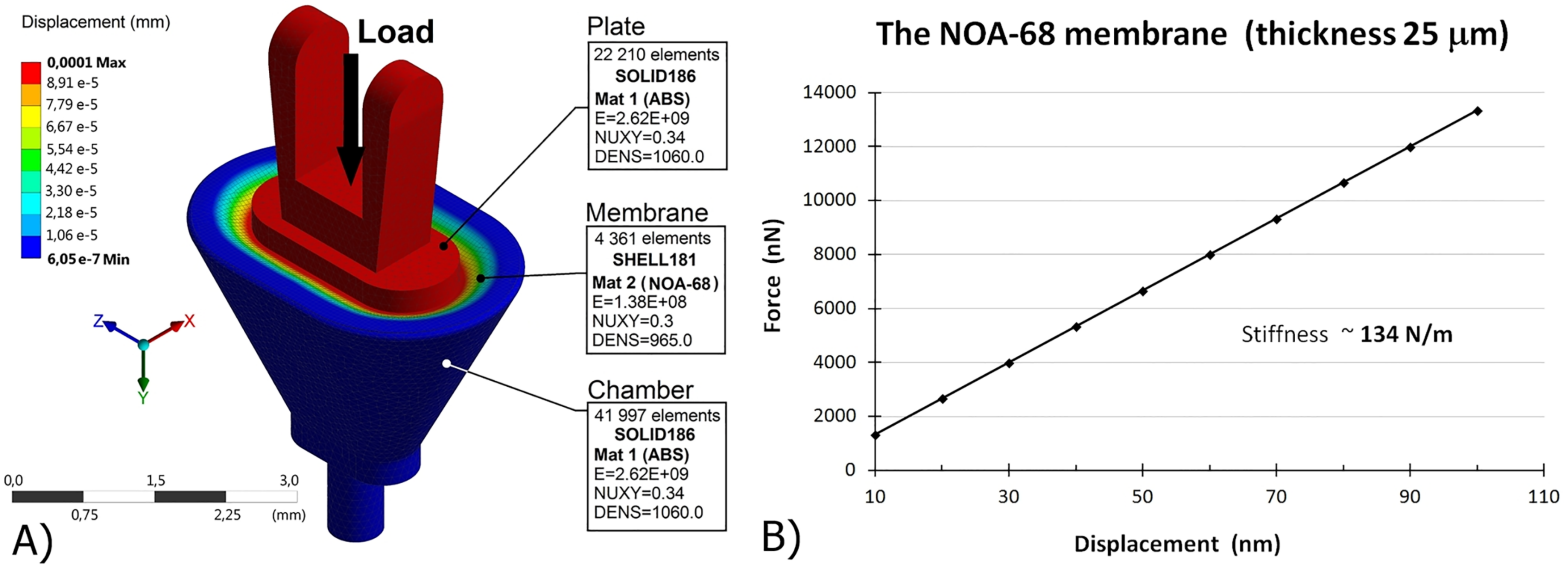
The FE model of the ChSP. (A) The FE model of the ChSP with displacement (black narrow) applied to the plate. (B) The force-displacement characteristic obtained as a result of the FE simulation for the NOA-68 membrane with thickness of 25 um.

In our prototype, the NOA-68 membrane had a thickness of ~25 um. We determined the membrane stiffness based on finite element (FE) simulation using ANSYS R17.0. The FE model consists of the plate, the 25-um membrane and the chamber [Fig 2(A)]. The geometry was taken from the 3D CAD model.

Since the membrane deflections during the sound transmission are small (max.100 nm), the membrane was modeled with linear material properties defined by the Young’s modulus E = 137.9 MPa (based on the manufacturer’s data https://www.norlandprod.com/literature/68tds.pdf) and the Poisson’s coefficient ν = 0.3. The chamber and the rigid plate were made from ABS-M30i (Acrylonitrile-Butadiene-Styrene). Their Young’s modulus was E = 2.62 GPa and the Poisson’s coefficient ν = 0.34. The membrane was meshed with 4361 shell elements (SHELL181) with a thickness of 25 um. Both the chamber and the plate were meshed with second order tetrahedral elements (SOLID186). The basis of the chamber was fixed in the space by constraining all degrees of freedom (DOF). The translational DOF of the plate were constrained only in X and Z directions. Displacements were successively applied to the plate surface in ten equal steps (each step 10 nm, a maximum displacement of 100 nm). The reaction force at the chamber base was calculated. As a result, we obtained the force-displacement characteristic [see Fig 2(B)]. Based on this characteristic, we determined the 25-um-membrane stiffness of about 134 N/m. This value is slightly higher than the AL stiffness (120 N/m), measured in human temporal bone [31].

Fig 1B shows the ChSP prototype. We implanted this prototype in the human temporal bone and measured the vibration of both the RW membrane and the ChSP plate.

### Implantation procedure

The management of human data was performed in accordance with Polish laws concerning the use of de-identified post-mortem human material.

**Fig 3:**
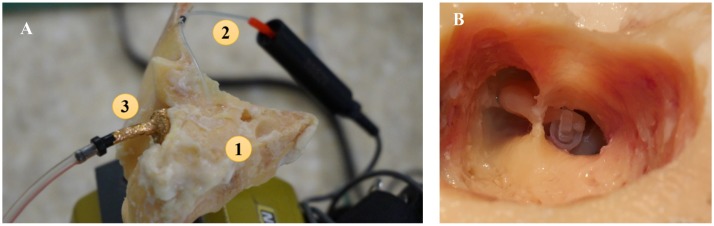
Experiment set up. (A) The specimen used in our experiments. 1 –human temporal bone, 2 –ER7-14C microphone tube with the attached microphone, 3 –ER3-26A foam ear tip with ER3-04 adapter. (B) The ChSP prototype implanted in the human temporal bone.

The temporal bone was collected from a human corpse selected in the Forensic Medicine Institute of Warsaw Medical University two days after death, according to standard practice developed by Schuknecht [32]. The middle ear condition was examined to confirm that no otosclerosis was present in the OW niche. The temporal bone was dissected under an operating microscope, using a standard set of micro-otosurgical equipment and a saw blade mounted on a dentist drill tool. Next the extended attico-antro-mastoidectomy and the wide posterior tympanotomy were performed. Part of the facial nerve canal, with the 7th cranial nerve, was removed to aide visibility of the OW niche. Mobility of the ossicular chain and the RW membrane response were both checked. Following this, a small hole (diameter ~1 mm) was made in the anterior wall of the external ear canal (EAC). Into this hole, the microphone tube (ER7-14C, Etymotic Research, Elk Grove Village, IL, USA) was inserted [see Fig 3(A)]. The tube end was positioned at a distance of approx. 2 mm from the tympanic membrane. Then the foam eartip (ER3-26A) with the adapter (ER3-04) was fixed in the shortened EAC.

In the first stage of the experiment, the RW vibration in the normal ear was measured. Immediately after this measurement, the stapedotomy with the ChSP prototype was undertaken. The SF was immobilized with glass-ionomer cement and the stapedial muscle was cut. The stapes suprastructure was removed and a 0.6-mm hole was drilled in the SF. Into this hole, the thin tube of the ChSP was inserted. The long process of the incus was fixed between the sidewalls of the RP [see Fig 3(B)]. Finally, the hole in the SF was sealed by a blood clot and the chamber was fixed to the OW niche using glass-ionomer cement. After the ChSP-stapedotomy was complete, the second stage of the experiment was conducted.

### Measurement procedure

The vibrations of the RW membrane were measured in the human temporal bone specimen before and after the ChSP-stapedotomy using a scanning laser-Doppler vibrometer (SLDV). The measurement procedure was similar to that described in [10]. Briefly, an acoustic signal (90dB SPL, 0.8–8 kHz) was introduced to the EAC via a loudspeaker (ER-2, Etymotic Research, Elk Grove Village, IL, USA) attached to an adapter (ER3-04). The 90-dB SPL near the tympanic membrane was controlled by a ER-7C microphone connected to the microphone tube (ER7-14C). RW vibrations were recorded using the SLDV (PSV400, Polytec GmbH, Waldbronn, Germany), before and after the ChSP-stapedotomy. Additionally, the vibrations of the ChSP plate were measured after the stapedotomy to compare them with the stapes vibrations in the normal ear.

### Data analysis

The recorded data was visualized using ScanViewer 1.4 software (Polytec GmbH, Germany). Vibration patterns of the cochlear input (the plate of the ChSP) and cochlear output (the RW membrane) were based on displacements measured at 177 and 155 scan points, respectively. In order to quantify the data, input ratio (IR) and output ratio (OR) parameters were calculated.

### Input ratio (IR)

In order to analyze the restoration of the ossicular chain mobility by the ChSP, and to compare it with the mobility of both the normal middle ear and the middle ear implanted with the 0.4-mm piston, we defined two IR parameters: IR_0.4piston_ and IR_ChSP_.

For the 0.4-mm piston prosthesis, the IR parameter (IR_0.4piston_) equals the displacement amplitude of the 0.4-mm piston relative to the displacement amplitude of the stapes head (SH) measured in the same ear before and after stapedotomy. The IR_0.4piston_ was calculated as IR_0.4piston_ = 20 log (d_0.4piston_ / d_SH_) where d_0.4piston_ and d_SH_ is the displacement amplitude of the 0.4-mm-piston and the stapes head, respectively. The IR_0.4piston_ was calculated based on data provided in Kwacz et al. [33].

Similarly, for the ChSP, the IR parameter (IR_ChSP_) equals the displacement amplitude of the ChSP plate relative to the displacement amplitude of the normal SH. The IR_ChSP_ was calculated as IR_ChSP_ = 20 log (d_ChSP_Plate_ / d_SH_) where d_ChSP_Plate_ is the displacement amplitude of the rigid plate (RP).

### Output ratio (OR)

To analyze the effectiveness of the cochlea stimulation by the ChSP, and compare it to stimulation by both the SF and the piston prosthesis, we defined two OR parameters: OR_ChSP_ and OR_0.4piston_.

For the ChSP, the OR parameter (OR_ChSP_) equals the volume displacement (VD) at the RW induced by the ChSP, relative to the VD at the RW induced by the normal SF in the same ear prior to the ChSP-stapedotomy. The OR_ChSP_ was calculated as OR_ChSP_ = 20 log (VD_RW_ChSP_/VD_RW_normal ear_) where VD_RW_ChSP_ is the VD at the RW after the ChSP-stapedotomy, and VD_RW_normal ear_ is the VD at the RW in the normal state.

Similarly, for the 0.4-mm piston prosthesis, the OR parameter (OR_0.4piston_) is the VD at the RW induced by the 0.4-mm piston, relative to the VD at the RW induced by the normal SF in the same ear before the piston-stapedotomy. The OR_0.4piston_ was calculated as OR_0.4piston_ = 20 log (VD_RW_0.4piston_ / VD_RW_normal ear_) where VD_RW_0.4piston_ is the VD at the RW after the piston-stapedotomy. The OR_0.4piston_ was calculated based on based on data provided in Kwacz et al. [33].

## Results

### Vibrations at the cochlear input

**Fig 4:**
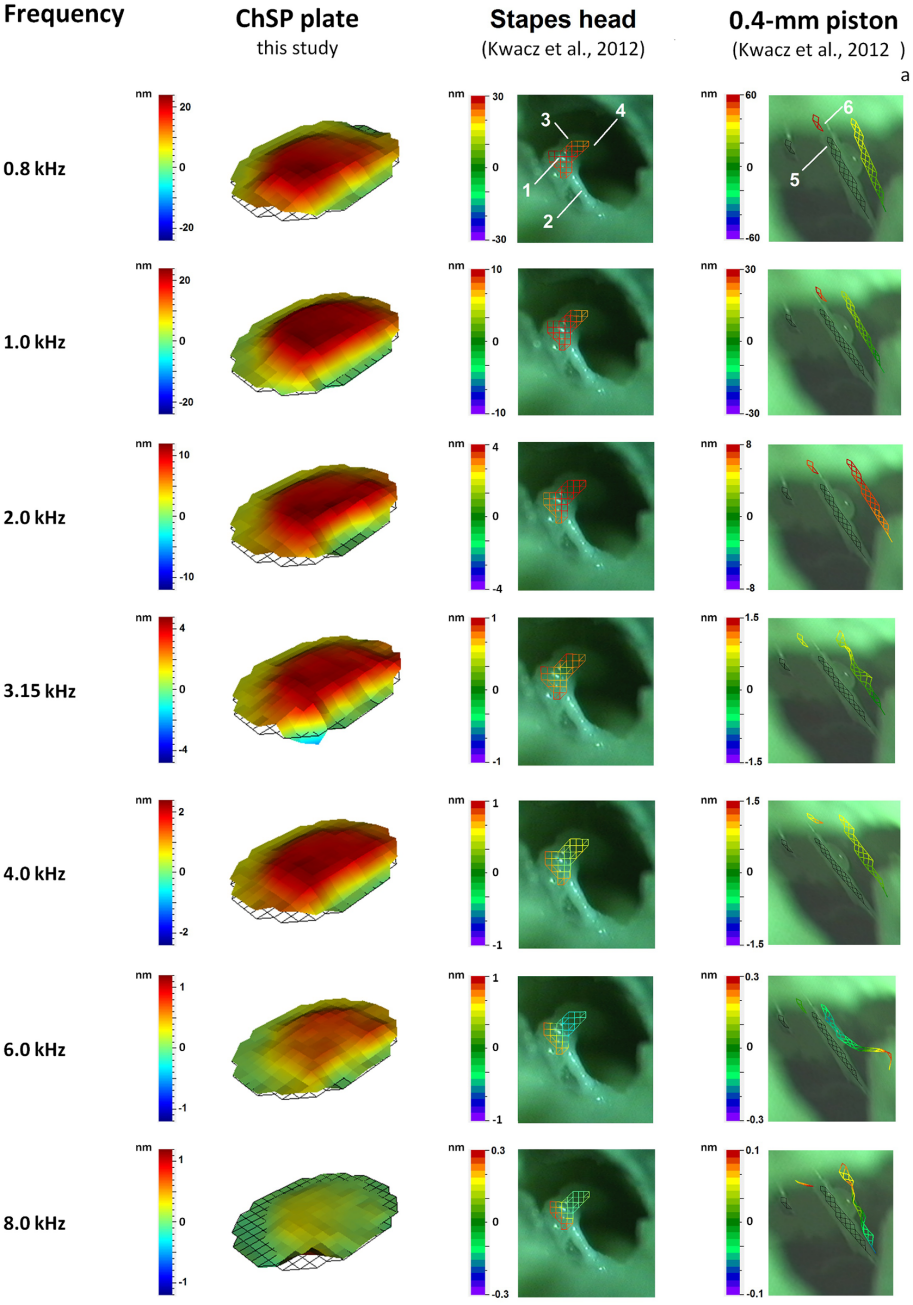
Three-dimensional visualization of displacements at the cochlear input. Left column–vibration of the ChSP plate, middle column–vibration of the normal stapes [33], right column–vibration of the 0.4-mm piston prosthesis [33]. All measurements for 90 dB SPL at the tympanic membrane. 1 –stapes head, 2 –tendon of the stapedial muscle, 3 –lenticular process, 4 –long process of the incus, 5 –piston, 6 –wire loop of the piston prosthesis.

Fig 4 shows the patterns of the ChSP plate vibrations at different frequencies (0.8–8 kHz).

**Fig 5:**
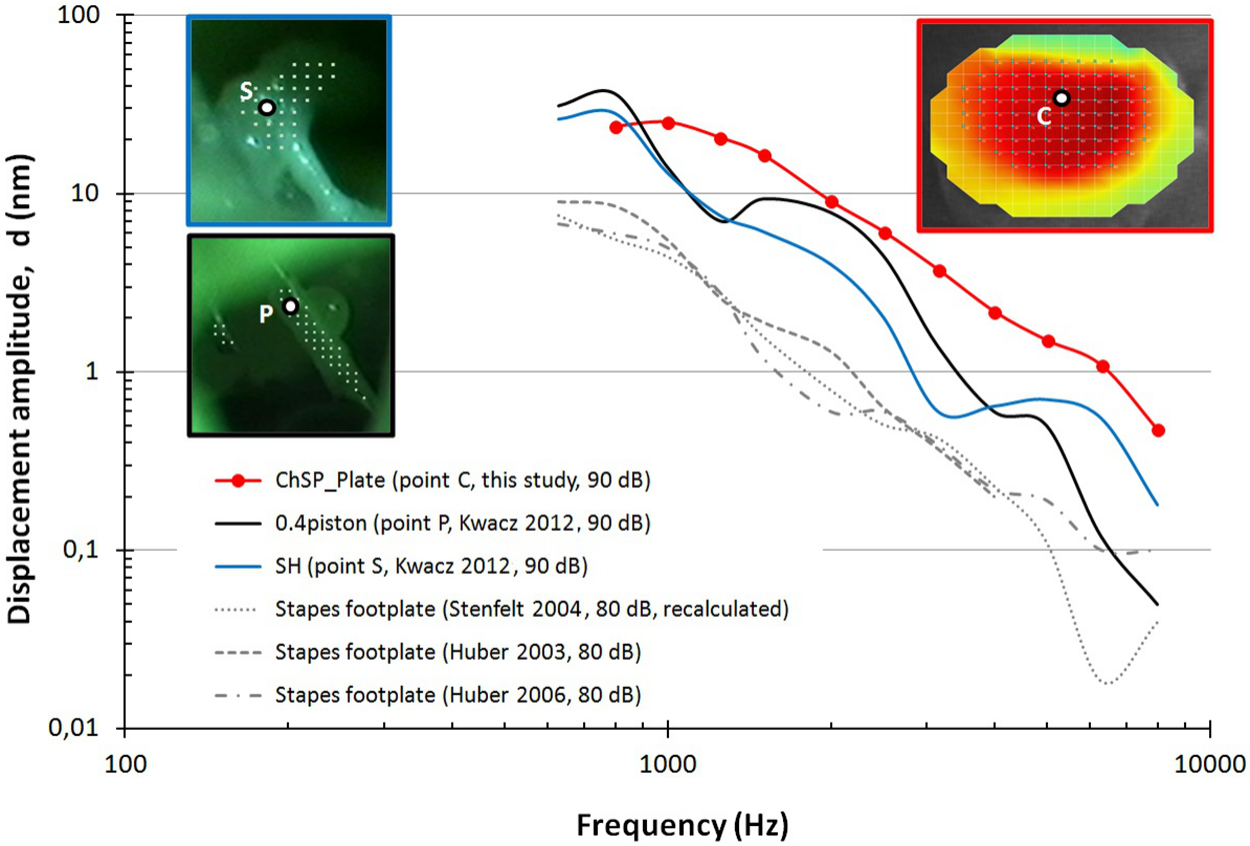
Displacement-frequency characteristics. The ChSP plate vibration (d_ChSP_Plate_, red line, point C, this study), the normal stapes head (d_SH_, blue line, point S, data from [33]), the 0.4-mm piston (d_0.4piston_, black line, point P, data from [33]), and literature data (stapes footplate, data from [34–36]).

The ChSP plate moves in a piston-like manner. Even at high frequencies, no rocking or hinging motion is observed. As the ChSP plate moves in this piston-like way, we analyzed the plate displacement amplitude (d_ChSP_Plate_) at a single point C, indicated in Fig 5 (picture surrounded by red line). The d_ChSP_Plate_ values at that point C for different frequencies are listed in Table 1 and the displacement-frequency characteristic is shown in Fig 5 (red line). The d_ChSP_Plate_ decreases from 2.3·10^−8^ m at 0.8 kHz to 4.7·10^−10^ m at 8 kHz. For 1.0 kHz, a slightly peak to 2.6·10^−8^ m is observed.

**Table 1:** Displacement amplitude at the cochlear input.

f (kHz)	d_ChSP_Plate_ (m)	d_SH_ (m)	d_0.4 piston_ (m)
**0.80**	2.34E-08	2.81E-08	3.61E-08
**1.00**	2.57E-08	1.30E-08	1.40E-08
**1.25**	2.06E-08	7.52E-09	7.03E-09
**1.50**	1.64E-08	6.10E-09	9.30E-09
**2.00**	9.06E-09	4.07E-09	7.82E-09
**2.50**	6.11E-09	2.02E-09	4.47E-09
**3.15**	3.75E-09	6.04E-10	1.39E-09
**4.00**	2.18E-09	6.42E-10	6.03E-10
**5.00**	1.50E-09	7.03E-10	5.01E-10
**6.00**	1.09E-09	5.51E-10	1.20E-10
**8.00**	4.74E-10	1.82E-10	5.00E-11

d_ChSP_Plate_−displacement of the ChSP plate (point C in Fig 5), d_SH_−displacement of the stapes head (point S in Fig 5, data from [33]), d_0.4 piston_−displacement of the 0.4-mm piston (point P in Fig 5, data from [33]). All measurements for 90 dB SLP at the tympanic membrane.

For comparison, Fig 5 shows displacements of the normal stapes head at a single point S (d_SH_, blue line), and displacements of the 0.4-mm piston at a single point P (d_0.4piston_, black line). For frequencies 1.25–4 kHz, the 0.4-mm piston vibrates with a higher amplitude than the normal stapes head. For frequencies above 4 kHz, the piston amplitude rapidly decreases. In contrast, the RP vibrates with significantly higher amplitude than the normal stapes head for all measurement frequencies. Only at 0.8 kHz is the d_ChSP_ close to the d_SH_.

The displacement curves for the normal stapes footplate shown in Fig 5 have a similar course to the d_SH_ curve with smaller values of displacement amplitudes. These smaller values reported in [34–36] result from 80 dB SPL at the tympanic membrane, which is 10 dB lower than in our measurement.

**Fig 6:**
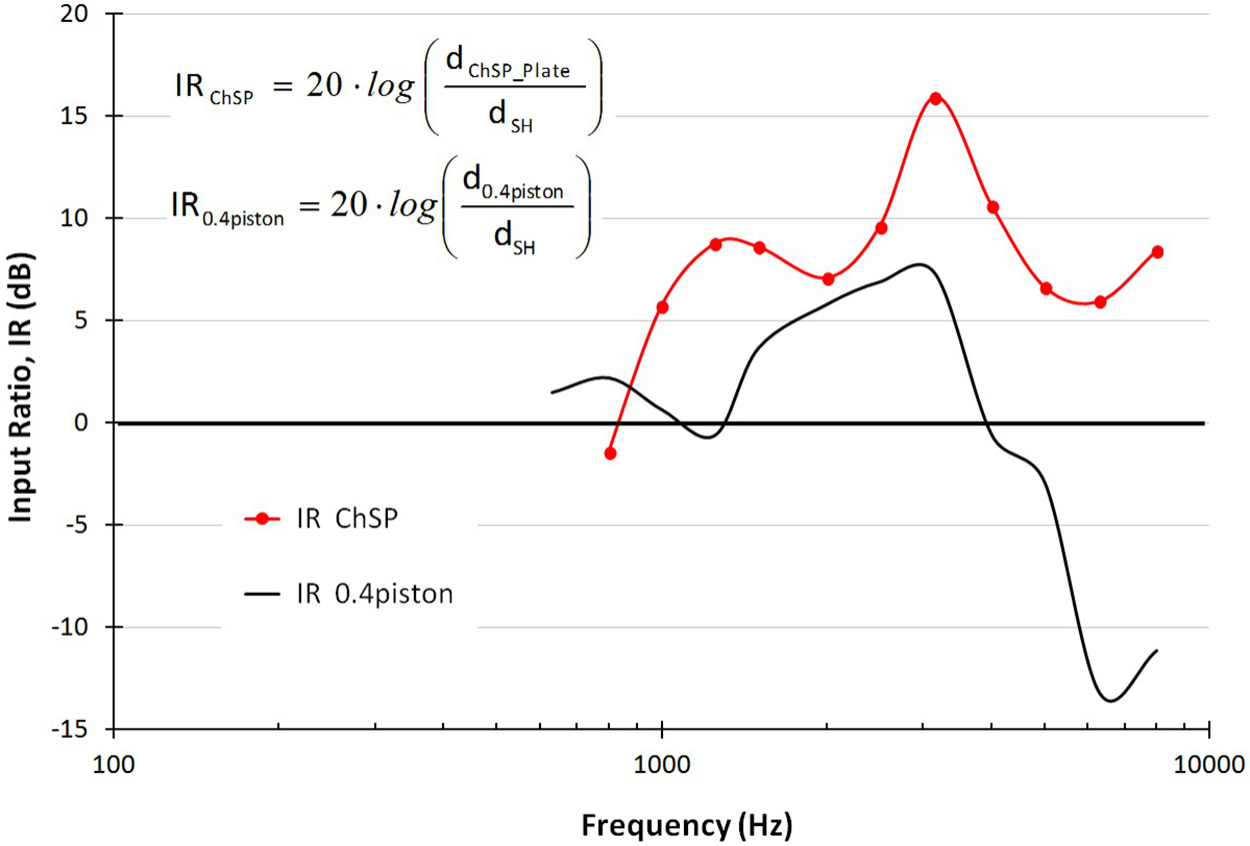
InputRatio-frequency characteristics. The ChSP (IR_ChSP_, red line) and the 0.4-mm piston (IR_0.4 piston_, black line).

Fig 6 shows the IR_ChSP_ (red line) and the IR_0.4piston_ (black line) in the 0.8–8 kHz frequency range.

The IR_ChSP_ values are mostly within the range of 5–10 dB. Only at 3.15 kHz is the RP amplitude significantly higher than the normal stapes amplitude (IR_ChSP_ = 16 dB). This corresponds to the sharp decrease in the stapes displacement amplitude measured in the normal ear (see Fig 5). The IR_ChSP_ curve indicates that the ChSP allows restoration of ossicular chain mobility above the physiological level for f > 1 kHz.

The IR_0.4piston_ values are mostly between 0 dB to 7 dB for frequencies up to 4 kHz. For f > 5 kHz, the piston mobility rapidly decreases and the IR_0,4piston_ reaches -13 dB at 6.3 kHz. The IR_0.4piston_ curve shows that the 0.4-mm piston allows restoration of ossicular chain mobility to the physiological level only for f < 4 kHz. This may result in insufficient hearing outcomes after piston-stapedotomy for f > 4 kHz.

### Vibrations at the cochlear output

**Fig 7:**
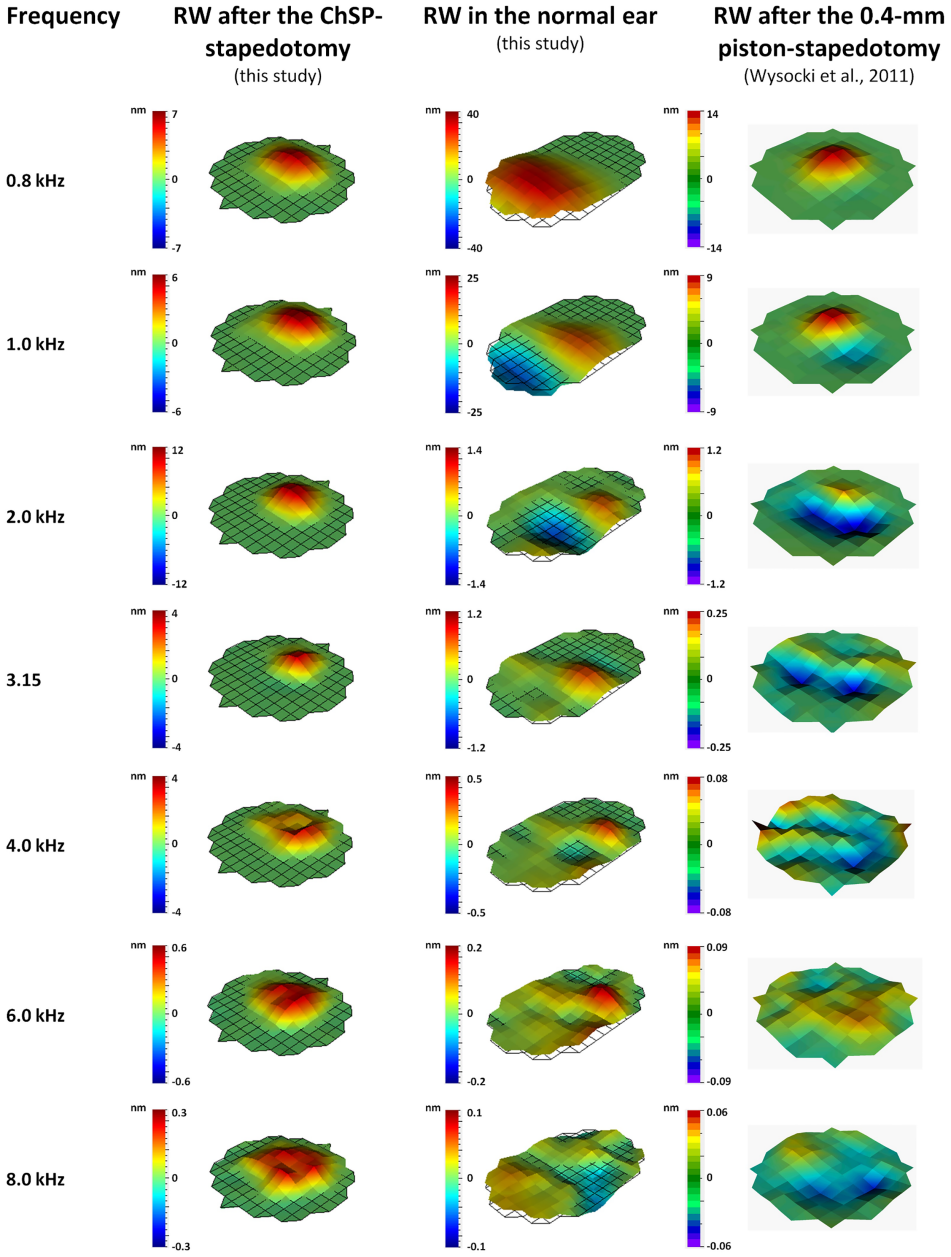
Three-dimensional visualization of displacements at the cochlear output (the RW membrane). Left column–RW displacement after stapedotomy with the ChSP (this study), middle column–RW displacement before stapedotomy (normal ear, this study), right column–RW displacement after stapedotomy with the 0.4-mm piston prosthesis (data from [10]). All measurements for 90 dB SPL at the tympanic membrane.

Fig 7 shows three-dimensional visualizations of the RW vibration, after the ChSP-stapedotomy (RW_ChSP_), at different frequencies (0.8–8 kHz). At all frequencies, the vibration shape is almost the same and the maximum amplitude occurs near the center of the RW membrane. For comparison, Fig 7 shows the RW vibrations both in the normal ear and after the 0.4-mm-piston stapedotomy. Below 1 kHz, in the normal ear, all points on the RW membrane vibrate in phase. This corresponds to the piston-like movement of the stapes. For frequencies 1–3 kHz, the RW membrane moves in two sections with a phase difference of about 180 degrees. This corresponds to the rocking motion [37] of the SF. Above 3 kHz, the RW vibrations are highly complex. This relates to the SF motion with both rocking and hinging components. Similarly, the shape of the RW vibration after the 0.4-mm-piston stapedotomy changes depending on the frequency. It was also observed that vibrations of the 0.4-mm piston are not piston-like for frequencies above 2 kHz (see Fig 3 in Kwacz et al. [33]).

**Fig 8:**
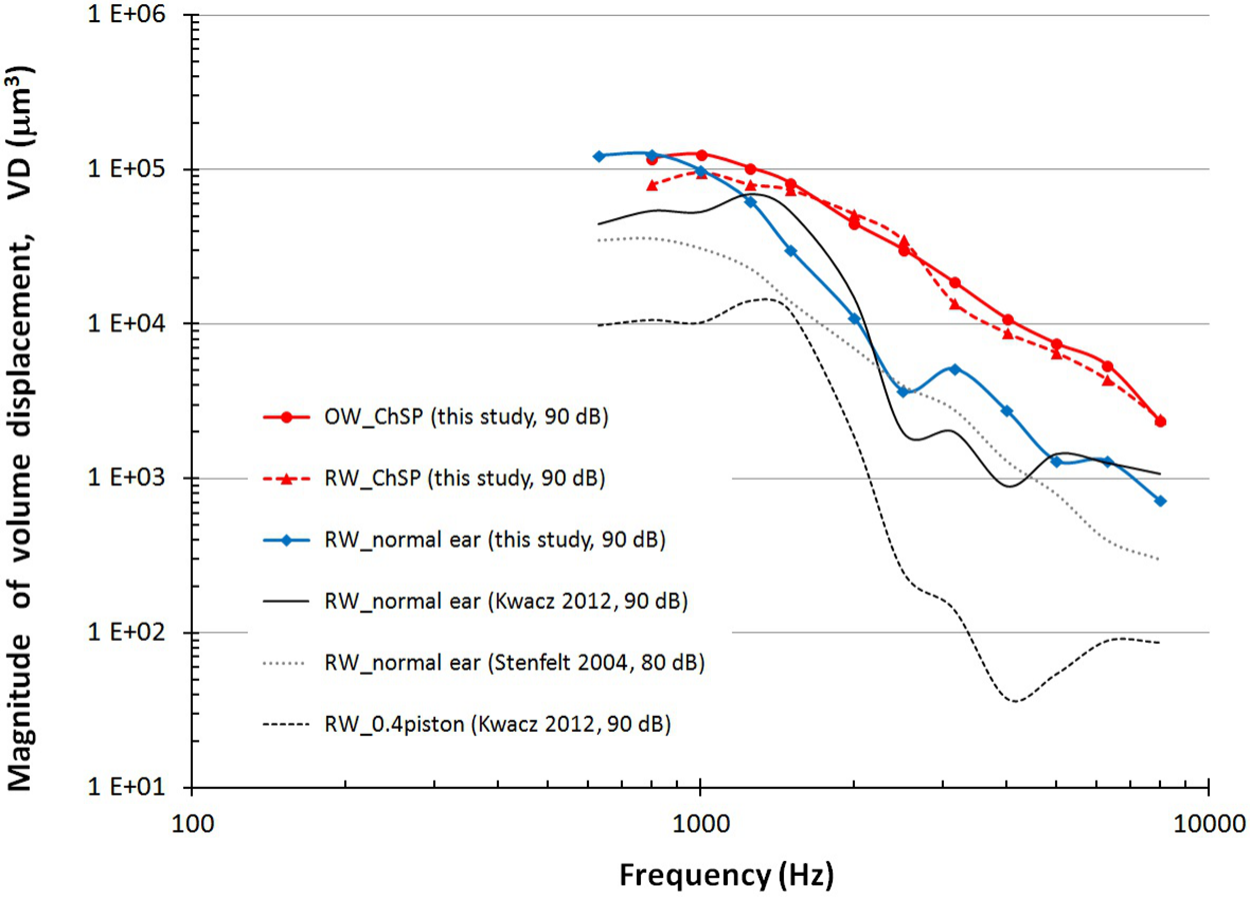
Volume displacement-frequency characteristics. Volume displacement-frequency characteristics at the RW after the ChSP-stapedotomy (RW_ChSP, red dashed line, 90 dB SPL, this study), in the normal ear (RW_normal ear, blue line, 90 dB SPL, this study; RW_normal ear, black line, 90 dB SPL, data from [33]; RW_normal ear, grey dotted line, 80 dB SPL, data from [34]), after the 0.4-mm piston-stapedotomy (RW_0.4 piston, black dashed line, 90 dB SPL, data from [33]). For comparison the volume displacement-frequency characteristic at the OW after the ChSP-stapedotomy (OW_ChSP, red solid line, 90 dB SPL, this study).

Based on the measured RW displacement amplitudes, the magnitude of volume displacement (VD) at the RW was calculated and is shown in Fig 8. For f ≥ 1.5 kHz, the VD at the RW induced by the ChSP (VD_RW_ChSP_, red solid line) is significantly higher than the VD at the RW induced by both the normal stapes (VD_RW_normal ear_, blue line) and the 0.4-mm piston (VD_RW_0.4piston_, black dashed line).

**Fig 9:**
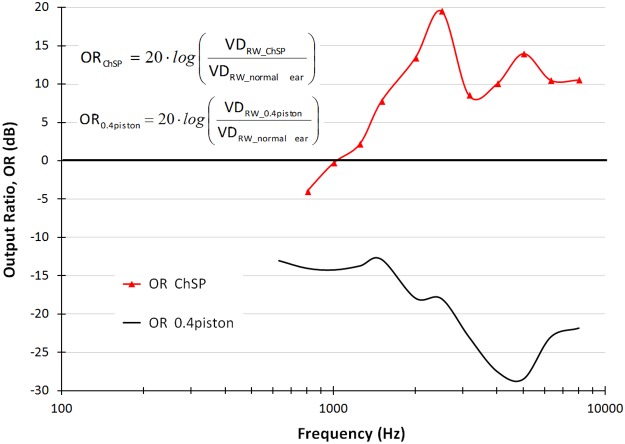
OutputRatio-frequency characteristics for the ChSP (OR_ChSP_, red line) and the 0.4-mm piston (OR_0.4 piston_, black line).

Fig 9 shows the OR_ChSP_ for the ChSP (red line) and the OR_0.4piston_ for the 0.4-mm piston prosthesis (black line).

The OR_ChSP_ values range between 0–20 dB for f ≥ 1 kHz. Only at 0.8 kHz is the VD at the RW induced by the ChSP, lower than the VD at the RW in the normal ear (OR_ChSP_ = -4 dB). This corresponds to the small IR_ChSP_ value (-1.4 dB for f = 0.8 kHz, Fig 6). The OR_ChSP_ curve indicates that the ChSP effectively stimulates the cochlea and allows restoration of cochlear macromechanics even above a physiological level.

The OR_0.4piston_ values are always lower than 0 dB. For f ≤ 2.5 kHz, the OR_0.4piston_ is mostly between -10 dB and -20 dB. Above 3 kHz, the OR_0.4piston_ is rapidly declining, reaching values below -25 dB. The OR_0.4piston_ curve indicates that the 0.4-mm piston never restores the RW vibrations to a physiological level. This proves that the 0.4-mm piston ineffectively stimulates the cochlea.

## Discussion

Use of a piston to stimulate the cochlea is the most common method used to improve hearing in the otosclerotic ear. However, altered biomechanics of the middle ear leads to reduced perilymph stimulation [10, 11]. This results in lower BM vibration and lower excitation of the hair cells. In advanced otosclerosis, with severe mixed hearing loss, piston stapes prosthesis often does not result in satisfactory hearing improvement. An alternative option is cochlear implantation [38,39], the Vibrant Soundbridge (VSB) or the Codacs System (Cochlear Ltd., Sydney Australia), together with a piston prosthesis implantation [40,41]. However, all these procedures are expensive, complex and require lengthy operation times.

To overcome the unsatisfactory hearing results after stapedotomy or re-stapedotomy, in advanced otosclerosis as well, we propose a new ChSP that mimics functioning of the natural ear.

### New ChSP–Design and functioning

The proposed ChSP (Fig 1) consists of a fluid filled chamber in the shape of a truncated cone. At the smaller base of the cone, a thin tube is formed to insert the ChSP into the SF hole. The tube allows free fluid flow between the chamber and the vestibule. After placing the tube into the hole, the fluid merges with perilymph. In this study, we used deionized water to fill the chamber. Using deionized water can disrupt the electrolyte balance of the perilymph. This was immaterial in our experiment, but poses risks to electrochemical processes in the living cochlea. As the perilymph is a liquid with high Na^+^ and low K^+^ concentration, so a liquid with a similar electrolyte concentration should be used in a prosthesis intended for clinical use.

The vibrations of the rigid plate are converted to a sound pressure in the fluid-filled chamber. Since the fluid can be considered incompressible and the chamber wall infinitely stiff, the fluid volume displacement at the rigid plate and at the open end of the tube should be equal. This volume displacement generates a pressure wave into the cochlea and the pressure wave elicits travelling waves on the BM. To restore normal hearing after the ChSP-stapedotomy, the fluid volume displacement (VD = area×displacement) produced by the rigid plate should be the same as that produced by the normal SF. To maintain this requirement, the stiffness of the flexible membrane should be close to stiffness of the annular ligament (AL) (~120 N/m, [31]).

ABS-M30i is a rigid and biocompatible material suitable for micro-3D printing. The critical chamber dimension is the wall thickness and the inner diameter of the thin tube that allows fluid contained in the chamber to merge with the perilymph. We assumed that the tube outer diameter should be comparable to the piston diameter. In our ChSP prototype, the outer diameter of the tube was 0.5 mm [see Fig 1(A)]. It is commonly accepted, that making a 0.5-mm hole in the SF is permitted during stapedotomy. Due to the limitations of the ProJet 3D Printer, the wall thickness of the tube cannot be less than 0.1 mm. Thus, the inner diameter of the tube was ~0.3 mm.

### Implantation procedure and advantages of new ChSP

For the ChSP, the surgical technique remains the same as for the piston-stapedotomy, i.e. removing the stapes suprastructure, drilling a hole of 0.5–0.6 mm diameter in the SF, placing the prosthesis into the hole, and attaching it to the incus. However, the ChSP has several advantages.

Firstly, in the classical piston-stapedotomy, the piston prosthesis must be an appropriate length. Incorrect length is one of the main causes of stapedotomy failure [42]. A piston that is too short can be lifted out of the SF hole (i.e. by sneezing). A piston that is too long inserted into the vestibule can impale the structures of the membranous labyrinth, causing vertigo (i.e. in a retraction of the tympanic membrane). In an extreme case, a piston that is too long may cause direct trauma to the saccule and damage the inner ear. The desired piston length for each patient is determined intraoperatively by measuring the distance from either the medial or the lateral side of the incus to the SF. The piston should then be cut to the correct length. The proposed ChSP overcomes problems associated with prosthesis length because the chamber is immovable and the thin tube inserted to the SF hole does not vibrate. Therefore, the ChSP can not be lifted out of the hole nor impale the inner-ear structures.

Secondly, the classical piston-stapedotomy affects ossicular motion and the cochlear stimulation level. This is primarily due to the SF/piston area ratio. The piston area is 0.12–0.28 mm^2^ (for diameter of 0.4–0.6 mm, respectively) and is much smaller than the ~3-mm^2^ area of the normal SF. Our new ChSP mimics the natural stapes-vestibule interface. The flexible membrane (FM) mimics the stapes annular ligament (AL), the rigid plate mimics the normal stapes, and the fluid filled chamber acts as an additional compartment of the natural vestibule. The area of the large chamber base and rigid plate is 6.7 mm^2^ and 2.8 mm^2^, respectively. Such a design allows for a comparable level of cochlear stimulation by the ChSP, as by the normal stapes.

Thirdly, to restore normal hearing, the fluid volume displacement (VD) at the OW produced by stapes prosthesis, should be close to the fluid VD at the OW produced by the normal SF. The piston prosthesis does not meet this requirement. The ChSP gives the fluid VD comparable to the normal stapes because the area of the rigid plate is close to the area of the normal SF and the membrane stiffness is comparable to the AL stiffness.

Fourthly, in a classical piston-stapedotomy, it is particularly difficult to maintain concentricity between the piston and the hole made in the SF. Misalignment can lead to increased adhesion between the piston and the hole wall. In an extreme case, misalignment may result in mechanical friction between the piston and the wall, or could even cause immobilization of the piston. In such cases, the postoperative hearing results are unsatisfactory and revision surgery is necessary. Our new ChSP does not require concentricity. The chamber does not vibrate and the thin tube inserted into the hole allows fluid to flow between the chamber and the vestibule. Even if the thin tube is not concentrically inserted vibrations are still properly transmitted.

Fifthly, after a piston-stapedotomy high frequency tinnitus can remain unresolved, even after closure of the ABG at tinnitus frequencies [43]. Some authors report worsening of tinnitus in 1–11% of patients [44–46], and declining high-frequency hearing, after stapes surgery [47]. This may be caused by surgical trauma of sensory hair cells due to sound generated by drilling and stimulation of the perilymph by the piston being too low. Since the ChSP generates a much greater stimulation, compared to the piston, it can create an effective masking effect that may help patients with high-frequency tinnitus.

### Comparison of vibrations before and after stapedotomy

Fig 4 shows the ChSP plate vibration for frequencies 0.8–8 kHz. There is a piston-like vibration shape for all measurement frequencies. Above 2 kHz, the ChSP plate motion significantly differs from the healthy SF motion, which shows some level of rotation [37]. There are two possible reasons for this: (1) the smaller distance between the incus and the plate (~1.5 mm), compared to that between the incus and the SF (~4.5 mm). Due to the 1.5-mm distance, even complex motion of the incus will not result in rotation of the ChSP plate. (2) The symmetrical geometry of the ChSP. The shape of the ChSP plate is regular, in contrast to the shape of the healthy SF. Moreover, the plate is centrally located on the flexible membrane with a constant thickness. In a healthy ear, neither the thickness nor width of the AL are constant [48]. The observed piston-like vibration of the ChSP plate gives a larger VD at the OW, compared to the VD generated by the healthy SF, especially for frequencies above 2 kHz.

To assess the ossicular chain mobility after a stapedotomy, we analyzed the Input Ratio-frequency characteristics (see Fig 6). If the IR = 0 then the movable element vibrates with the same amplitude as the stapes, and ossicular chain mobility is restored to a physiological level. However, restoring the chain mobility does not imply effective cochlear stimulation. Effective stimulation depends on the volume displacement (VD), rather than on the displacement amplitude at cochlear input. Since the VD_RW_0.4piston_ is significantly lower than the VD_RW_normal ear_ (see Fig 8), the hearing results after piston-stapedotomy can be unsatisfactory and the postoperative ABG may be not completely closed, even though IR_piston_ ≥ 0. Our new ChSP, with the RP area similar to the SF area, will allow for the complete closure of the ABG for frequencies up to 10 kHz.

In Fig 7, we compared vibration shapes at the cochlear output (the RW membrane vibration) for three types of cochlea stimulation: (1) by the ChSP plate, (2) by the normal stapes, and (3) by the 0.4-mm piston. The vibration shape of the RW membrane after the ChSP-stapedotomy is almost identical for all measurement frequencies. The reason for this is the piston-like motion of the ChSP plate (see Fig 4).

We assessed the cochlear macromechanics after stapedotomy, and analyzed the effectiveness of the cochlea stimulation by different prostheses using the OR parameters (see Fig 9). If the OR = 0 then the RW membrane after stapedotomy vibrates with the same amplitude as the RW membrane in the normal ear. This means that the prosthesis effectively restores the cochlear macromechanics. Therefore, the postoperative hearing results can be expected to be at a physiological level.

## Conclusions

New chamber stapes prosthesis (ChSP) is dedicated to assisting patients with stapes otosclerosis. The design and functioning of the fluid-filled ChSP mimics the natural stapes-vestibule interface. The new prosthesis provides similar fluid VD at the otosclerotic OW as the fluid VD at the normal OW. A ChSP-stapedotomy is performed in the same way as a piston-stapedotomy. However, a ChSP-stapedotomy is safer for patients because it does not require the insertion of a movable element into the vestibule. Based on our preliminary experimental results, hearing results after a ChSP-stapedotomy will be better than after a piston-stapedotomy, especially for frequencies above 1.5 kHz. Further studies are necessary to optimize geometry and develop appropriate technology for the ChSP-flexible membrane fabrication.
